# Social identity continuity and mental health among Syrian refugees in Turkey

**DOI:** 10.1007/s00127-017-1424-7

**Published:** 2017-07-21

**Authors:** Anouk Smeekes, Maykel Verkuyten, Elif Çelebi, Ceren Acartürk, Samed Onkun

**Affiliations:** 1 Ercomer, Faculty of Social and Behavioural Sciences, Utrecht University, Padualaan 14, 3584 CH Utrecht, The Netherlands; 2 Sehir University, Istanbul, Turkey

**Keywords:** Identity continuity, Mental health, Well-being, Refugees, Turkey

## Abstract

**Purpose:**

Building upon social psychological work on social identity and mental health, this study among Syrian refugees in Turkey examined the importance of multiple group memberships and identity continuity for mental health and well-being.

**Method:**

A survey study was conducted among the very difficult to reach population of Syrian refugees (*N* = 361). With path analysis in AMOS the associations were examined between multiple group memberships, social identity continuity and mental health and psychological well-being.

**Results:**

Indicate that belonging to multiple groups before migration was related to a higher likelihood of having preserved group memberships after migration (i.e., sense of social identity continuity), which, in turn, predicted greater life satisfaction and lower levels of depression. Multiple group membership, however, was also directly related to higher depression.

**Conclusions:**

Findings are discussed in relation to the importance of multiple group membership and feelings of identity continuity for refugees.

## Introduction

It is estimated that around 11 million Syrians have fled their homes since the outbreak of the civil war in March 2011 [1]. While the majority is internally displaced, many of them have fled to neighboring countries, such as Turkey. Although Turkey has hosted large numbers of Syrian refugees (about 3.1 million), many of them are left without effective protection or access to jobs and services, such as health care [2]. These refugees not only have to deal with the acute stressors of war, but also often face post-migration challenges, such as marginalization, acculturation problems, socioeconomic disadvantage and ‘cultural bereavement’ [3, 4], which negatively impact their well-being. Specifically, it is found that refugees often experience a wide variety of both physical and mental health problems, such as depression, anxiety and post-traumatic stress disorder [5, 6].

Research indicates that there also are factors that can protect refugees’ mental health and well-being, such as a stronger sense of ethnic group belonging [7]. While there is much evidence that people can draw on social bonds to maintain and enhance their health and well-being, very few studies have examined the role of a sense of group belonging as a protective factor for refugees’ mental health and well-being. Group belonging with its associated sense of social identity may function as a protective factor for refugees, constituting an important source of mental and physical health in the face of difficulties [8]. Furthermore, having multiple group identities has been found to buffer the negative consequences associated with identity change and identity loss [9]. People who only have one or a few group memberships are more vulnerable to negative life transitions, compared to individuals whose self-definition incorporates a greater number of group memberships [10]. One important reason is that individuals with multiple group memberships have a greater likelihood of maintaining a sense of belonging to some of these memberships following a major life transition, which subsequently enhances their mental health and well-being [9]. In other words, having multiple group memberships helps people to maintain a sense of social identity continuity. There is a growing body of work in social psychology that shows that perceived identity continuity is an important factor for mental health and well-being [11–13]. In the current study, we focused on Syrian refugees and examined whether having multiple group memberships before migration helps these people to maintain a sense of identity continuity after migration, and is thereby positively associated with mental health and well-being.

### Social identity continuity and well-being

Self-continuity refers to having a sense of connection between one’s past and present self and is found to be positively related to mental health and well-being [14]. For instance, studies demonstrate that temporal stability in one’s self-evaluations is linked to higher levels of positive effect and lower levels of depression [15, 16]. Conversely, a sense of self-discontinuity is associated with lower mental health, such as feelings of dissociation [12], distress [17], and even suicide [18].

People do not only experience a sense of (dis)continuity of their personal self but are also able to perceive their ‘social self’ as temporally enduring [13, 19, 20]. This notion is based on the social identity perspective [21] which argues that people derive a sense of self from their memberships in groups. As such, people are able to experience a sense of social identity continuity. Work by Sani et al. [13] demonstrated that perceptions of social identity continuity are related to a stronger sense of well-being, and Haslam et al. [9] found that social identity continuity predicted well-being following a stroke. This line of work is embedded in a larger literature demonstrating that a sense of social identity has beneficial effects on mental health and well-being because of the feelings of certainty, meaning, relatedness, esteem, and efficacy that social identities provide [22, 23]. Part of this literature focuses on how social identity can help people to cope with negative life transitions and identity change [24].

### Maintaining social identity continuity after a negative life transition

There is much evidence that undergoing negative life transitions, such as losing one’s job or one’s home, has a detrimental impact on mental health and well-being [25, 26]. Research on coping with these transitions often takes an individual differences perspective by studying the personality traits that help individuals to deal with these hardships [27]. Recently, studies have focused on how social identity can help to foster or maintain mental health and well-being in the context of negative life transitions [28, 29]. This line of work shows that a crucial factor in maintaining mental health and well-being after such transitions is the number of group memberships before the transition [9, 30, 31]. Belonging to multiple groups is found to be related to lower chances of illness, depression, distress and stronger feelings of positive affect [32–36].

One reason why multiple group memberships might have these beneficial effects is that it increases the likelihood of being able to maintain a feeling of belonging to some of one’s group memberships after the negative life transition, hence enabling a sense of social identity continuity [24]. However, this proposition has so far only been examined in a single study among people who had a stroke [9]. This study showed that multiple group memberships before the stroke protected well-being because this resulted in a greater likelihood of maintaining some of these group memberships after the stroke. The current research examines whether similar processes may be at work for people whose negative life transition involves leaving one’s country and (often) friends and relatives behind to escape from war. Specifically, we focus on Syrian refugees who fled to Turkey and examine whether having multiple group memberships before migration protects their well-being after migration, because it relates to a higher likelihood of maintaining a sense of group memberships after migration.

### Syrian refugees in Turkey

The study was conducted in a unique context of Syrian refugees in Turkey. Turkey has declared an open door policy for refugees from Syria and provides them a “temporary protection” since April 2011. Currently, there are around 3.1 million Syrian refugees in the country, making Turkey the largest host of refugees in the world [37]. However, Turkey does not grant Syrians “refugee status” which would imply legal rights, but only grants a temporary asylum seeker status. It is estimated that almost 90% of the refugees live outside refugee camps in rural and urban areas, in extremely challenging conditions with limited access to basic services. The ever growing number of refugees living outside the camps makes the organization of relieve and support programs difficult to implement. Syrian refugees face major challenges, for example, in finding housing and work, or they have to work under very poor conditions and for low wages. Additionally, in many parts of the country there are strong intergroup tensions in communities hosting Syrian refugees. Public surveys indicate that anti-Syrian sentiments are common with 86% of the Turkish people wanting the government to stop the intake of refugees and 30% supporting the view that refugees should be sent back to their home country [38].

## Methods

### Participants and procedure

In 2015, a total of 361 Syrian people aged between 18 and 75 (*M*_age_ = 32.50, SD = 11.86) participated in the study; 42.1% was female and 56% was male (1.9% missing). The sample consisted of people with different educational levels: 28.5% graduated from primary school, 22.4% completed secondary school, 30.8% completed high school, and 18.4% completed a Bachelor’s degree and higher educational levels. The data were collected in two different cities in Turkey: one in the West (Istanbul) and one in the Southeast (Antep) where some of the highest numbers of refugees live outside the camps (Istanbul, 400,000, and Antep, 350,000). The completion of the questionnaire took about 25–30 min and participants gave informed consent to participate in the study.

All the questions were translated and back-translated to Arabic from English by two native Arabic speakers, except for the mental health measure for which an established Arabic translation is available. Five Syrian research assistants were trained in data collection and administered the survey to the participants in Arabic and in their homes. If the participants needed help with reading, the research assistants read the questions to the participants and recorded their answers.

## Measures

### Belonging to multiple groups before migration

We assessed the extent to which participants felt to belong to multiple groups before migration. Because we wanted to keep the questionnaire as short as possible and did not want to burden the refugee respondents too much, we used two of the original four items of Haslam et al. [9]: “I was a member of lots of different social groups in Syria”, and “I had friends who are in lots of different social groups in Syria”. The two items were highly correlated (*r* = 0.66, *p* < 0.001) and were combined into a scale. The response scale ranged from 1 (strongly disagree) to 5 (strongly agree).

### Continuity group memberships after migration

Four items (*α* = 0.78) were used to assess whether participants had the feeling that they had maintained their pre-migration group memberships after migration [9]. These items (5-point response scales) were: “After immigration, I still belong to the same groups I was a member of before”, “After immigration I still join in the same group activities as before”, “After immigration I am friends with people in the same groups as I was before”, and “After immigration, I continue to have strong ties with the same groups as before”.

### Life satisfaction

It was assessed using the well-known satisfaction with life scale [36]. Five items (7-point response scales; *α* = 0.80) were used and participants were asked about how satisfied they were with their life after migration.

### Mental health

We used the Hopkins Symptoms Checklist 25 (HSCL-25) [39] to measure how frequently participants experienced anxiety (10 items; *α* = 0.85) and depression (15 items, *α* = 0.81). HSCL-25 is a widely employed and cross-culturally validated measure that has also been used to investigate the mental health of immigrants and refugees [38, 39]. The response options were: 1 *(*not at all), 2 (a little), 3 (quite a bit) and 4 (extremely).

### Controls

We used age (in years), gender (1 = male, 0 = female), location of data collection (1 = Istanbul, 0 = Antep), and highest obtained educational level (1 = primary school, 2 = middle school, 3 = high school, 4 = Bachelor degree, 5 = Master degree, and 6 = Ph.D. degree) as control variables in the analyses.

## Results

### Mean scores and intercorrelations

The mean scores and intercorrelations between the key variables of interest are shown in Table 1. The mean scores for multiple group memberships and continuity of group memberships after migration were relatively low and significantly below the neutral midpoint of the 5-point scale [*t*(351) = −5.63, *p* < 0.001 and *t*(346) = −15.97, *p* < 0.001, respectively]. This shows that people on average did not indicate to have many group memberships before migration and that they also found it difficult to maintain a sense of group belongings after migration. The mean score for life satisfaction was also significantly below the neutral midpoint of the 7-point scale [*t*(352) = −8.80, *p* < 0.001], indicating that participants were not very satisfied with their life after migration. Moreover, participants reported to suffer from mental health problems somewhat, but the mean scores for anxiety and depression were significantly below the midpoint of the 4-point scale [*t*(358) = −13.23, *p* < 0.001 and *t*(346) = −7.75, *p* < 0.001, respectively].

**Table 1:** Means, standard deviations and bivariate intercorrelations for all measures

	*M*	SD	1	2	3	4	5
Multiple groups before migration	2.55	1.51	–	0.34***	0.06	0.07	0.01
Continuity after migration	2.02	1.14		–	−0.01	−0.11*	0.30***
Anxiety	2.04	0.66			–	0.62***	−0.23***
Depression	2.24	0.64				–	−0.38***
Life satisfaction	3.24	1.59					–

*** *p* < 0.001, * *p* < 0.05

The correlations indicate that multiple group memberships before migration were positively correlated with identity continuity after migration, but not with anxiety, depression and life satisfaction. Continuity after migration was not related to anxiety, but correlated negatively with depression and positively with life satisfaction. Anxiety and depression were strongly and positively correlated with each other and both correlated negatively with life satisfaction.

### Path analysis

**Fig. 1:**
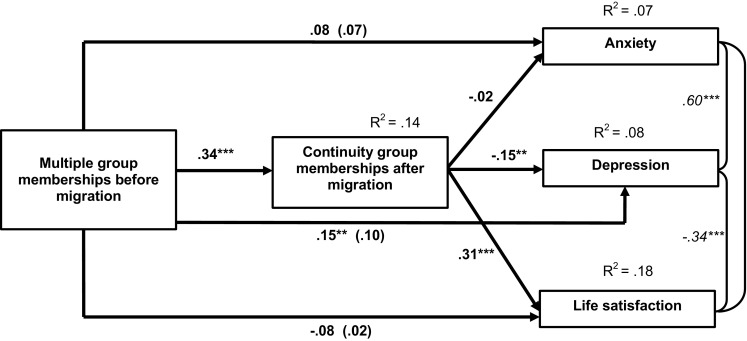
Structural equation model: Influence of multiple group memberships before immigration on mental stress and life satisfaction, via sense of social identity continuity (controlling for age, gender, education, and location)

We conducted a path analysis in AMOS 22.0 to test our prediction regarding the effects of multiple group membership before migration on anxiety, depression and life satisfaction, via continuity of group memberships after migration.^1^ The model is specified in Fig. 1. In this model, we controlled for age, gender, education, and location of data collection, by adding paths between these variables and the mediator and the dependent variables and by correlating them with the independent variable of multiple group memberships. The standardized paths and explained variance (*R* ^2^) are shown in Fig. 1. These analyses revealed that multiple group memberships before migration positively predicted continuity of group memberships after migration. Continuity, in turn, was a positive predictor of life satisfaction and a negative predictor of depression, but was not significantly related to anxiety (see Fig. 1). There were no total effects of multiple group memberships before migration on any of the dependent measures. However, the direct effect of multiple group memberships on depression was positive and significant when the mediator was included in the model. This could mean that there are both positive and negative pathways of multiple group membership before migration on depression. A higher number of group memberships before migration might not only have positive mental health implications because it contributes to a sense of continuity, but can also imply stronger feelings of disruption and loss leading to more depressive feelings. We elaborate on this finding in “Discussion”.

Regarding the control variables; age was not related to any of the variables in the path model. Gender was related to life satisfaction (*β* = 0.16, *p* = 0.001) and anxiety (*β* = 0.11, *p* = 0.041) with females reporting higher scores than males. Education was related to anxiety (*β* = −0.12, *p* = 0.035) and depression (*β* = −0.15, *p* = 0.008), with higher educated reporting lower scores. Location of data collection was related to multiple group memberships before migration (*r* = −0.12, *p* = 0.027), life satisfaction (*β* = 0.17, *p* < 0.001), anxiety (*β* = −0.18, *p* < 0.001) and depression (*β* = −0.14, *p* = 0.006), with people in Istanbul (compared to Antep) reporting less group memberships before migration, higher life satisfaction and lower anxiety and depression.^2^

### Analysis of indirect effects

We tested the indirect effects of multiple group memberships (via continuity of group memberships after migration) on anxiety, depression and life satisfaction, using bootstrapping procedures in AMOS 22.0. This model also included all the control variables. We generated 10,000 random bootstrap samples with replacement from the dataset and tested the model with these samples. As expected, the analysis revealed a significant negative indirect effect of multiple group memberships before migration on depression, with a standardized estimate of −0.052 and a 95% bias-corrected confidence interval of −0.096 to −0.015. We also observed a significant positive indirect effect of multiple group memberships before migration on life satisfaction, with a standardized estimate of 0.106 and a 95% bias-corrected confidence interval of 0.066–0.157. The indirect effect via anxiety was, however, not significant (lower BC = −0.047, upper BC = 0.029). These results indicate that the more group memberships Syrian refugees have before migration the more likely they are to maintain some sense of these group memberships after migration, which subsequently relates to higher levels of well-being.

## Discussion

The present research was conducted among a unique sample of Syrian refugees in Turkey and examined whether the sense of belonging to multiple groups prior to migration is associated with a feeling of social identity continuity after migration which subsequently protects mental health and well-being. While there is a growing body of social psychological research on the beneficial mental health and well-being effects of having multiple group memberships [8], few studies have examined the role of social identity continuity in these relationships [9].

The current study provides evidence that refugees who felt to belong to multiple groups before migration were more likely to report that they had maintained a sense of belonging to some of these group memberships after migration. This feeling of social identity continuity was subsequently positively associated with mental health and well-being. While this relation was strong in the case of reported life satisfaction, it was weaker for depression and non-significant for anxiety. Furthermore, path analysis provided evidence that multiple group memberships before migration were indirectly related to higher levels of life satisfaction and lower levels of depression (but not anxiety) via stronger social identity continuity. These findings are in line with previous work among people who suffered a stroke [9], showing that multiple group memberships before a negative life transition can promote well-being afterwards because there is a greater likelihood that some of these group memberships are preserved psychologically.

An important contribution of the present paper is that it examines the relations between multiple group memberships, social identity continuity and mental health and well-being among a refugee population. Although there are various studies about mental health and well-being among refugees [4, 40] very little is known about social identity as a protective factor in coping with this major negative life transition in this population. Research has shown that ethnic group belonging can serve as a protective factor for refugees’ mental and physical health [7], and our study extends this line of work by demonstrating the protective role of multiple group memberships before migration in fostering social identity continuity and thereby mental health and well-being among refugees.

Our findings also diverge from previous work in some ways. First, while other studies among community samples have established a positive direct link between belonging to multiple groups and life satisfaction [9, 34–36], in our study these measures were only indirectly linked via a stronger sense of social identity continuity. This suggests that for refugees social identity continuity after migration forms a key mechanism in connecting multiple group memberships to greater life satisfaction. Second, while previous studies have found a negative or no direct relation between belonging to multiple groups and depression [33, 35, 41], our study showed a positive relation. Thus, belonging to multiple groups was related to reduced levels of depression via social identity continuity, the remaining direct effect was positive. This suggests that, among our refugee sample, having multiple group memberships before migration can also have negative consequences for depression after migration. One reason for this could be that, for some Syrian refugees, thinking back about the different groups they belonged to before fleeing their country is a painful reminder of the people they have left behind, resulting in a stronger sense of depression. Thus, having multiple group memberships before migration not only implies a greater likelihood of maintaining some of these group memberships after migration but also having “more to lose” when leaving the country behind. This means that belonging to multiple groups before migration can be a double-edged sword in that it increases the chance of maintaining a sense of continuity but also the possibility of losing important social ties. To examine these further, future studies could consider the type of groups people think about when they say that they belonged to many groups. Group memberships do not have similar meanings and might fulfill different psychological functions making it important to examine the content of the different groups left behind [9].

Furthermore, while our proposed theoretical model explained a reasonable amount of variance in the outcome measure of life satisfaction, this was much less the case for the mental health measures. No significant effects on anxiety emerged and the reported direct and indirect effects on depression were small. This suggest that, for Syrian refugees, having multiple group memberships before migration is important for maintaining a sense of everyday well-being but less for their mental health. On the one hand, this finding is in line with previous work among people who suffered a stroke, showing that multiple group membership before a negative life transition was strongly linked to life satisfaction (via social identity continuity) but not to chronic stress [9]. On the other hand, this finding goes against research showing that belonging to multiple groups is a strong protective factor against depression [34, 41]. A potential explanation for these different findings is that the role of multiple group memberships and social identity continuity in fostering mental health is different for populations that have experienced a major negative life transition (such as refugees and stroke patients) compared to those who have not had such experiences. Another explanation is that we did not assess multiple group identifications but rather asked respondents about their number of group memberships. Self-reported group membership does not necessarily means that one subjectively identifies with that group. Research among large community samples of adolescents and adults has found that multiple group identifications are not only associated with life satisfaction [36] but also with depression and mental health more generally [34, 35]. Thus, the mental health-related benefits or costs of former group memberships might depend on how strongly refugees identify with these groups [8].

## Limitations and future directions

Our research has several limitations that can be addressed in future studies. The first one is that we conducted a path analysis with cross-sectional data which means that we tested causal processes while being unable to make strong empirical claims about the proposed causal mechanisms in our model. However, since our measures assessed participants perception of their group memberships’ before and after migration it is not very likely that a reversed causal order applies to this relationship. It is more plausible to consider belonging to multiple groups before migration as an antecedent of group membership continuity after migration. Nevertheless, it is possible that post-migration well-being encourages people to maintain their group memberships, or to report more positively on this measure, rather than vice versa. It would be interesting for future work to study the proposed mechanisms using a longitudinal design, in which multiple group memberships are assessed before the negative life transition of fleeing one’s country, and social identity continuity and mental health and well-being at multiple time points after migration. However, the practical challenges for such a longitudinal research would be very substantial and almost impossible to address (e.g., interviewing people in a war zone before they flee the country).

Although we managed to collect a unique dataset among a relatively large sample of Syrian refugees, the sample inevitably is selective. It is very difficult to reach Syrian refugees and data were collected among Syrian refugees in their homes and not among those living inside refugee camps (this was not allowed by government officials). Conditions inside refugee camps are different and more severe and it is possible that the observed relationships differ somewhat for people living in refugee camps. Moreover, it is likely that the effects of belonging to multiple groups on social identity continuity and mental health and well-being depend on a number of other factors. For instance, studies have shown that having many group identities only leads to greater well-being if these identities are important and in harmony with each other, but leads to lower well-being when these identities are subjectively in conflict [32]. This means that it might be important to examine the type of groups people think about when mentioning the ones they belonged to before migration. Future studies should try to collect data among a wider sample of refugees and focus on potential moderators of the relationship between multiple group identities, social identity continuity and well-being.

## Conclusion

Studying Syrian refugees in Turkey, we found that having multiple group identities before migration can serve as a protective factor for refugees’ mental health and well-being because it relates to a greater likelihood of maintaining some of these group memberships after migration (i.e., a sense of social identity continuity). Yet, having multiple group identities might also be a burden because it might illicit stronger feelings of social disruption and loss. Our research is one of the few studies that contribute to our understanding of the relationship between sense of social identity and health in refugee populations. Overall, the findings indicate that maintaining social identity continuity can be an important factor for refugees’ mental health and well-being.

## Footnotes

1 We used manifest variables as the sample size was too small to estimate a structural equation model with latent variables. A model with latent variables could not comply with the standard of 10 observations per estimated parameter.

2 We also tested the path model without all the control variables and this yielded the same pattern of results which are available upon request.
